# Khat, a Cultural Chewing Drug: A Toxicokinetic and Toxicodynamic Summary

**DOI:** 10.3390/toxins14020071

**Published:** 2022-01-20

**Authors:** Bárbara Silva, Jorge Soares, Carolina Rocha-Pereira, Přemysl Mladěnka, Fernando Remião, on behalf of The OEMONOM Researchers

**Affiliations:** 1 UCIBIO—Applied Molecular Biosciences Unit, REQUINTE, Toxicology Laboratory, Biological Sciences Department, Faculty of Pharmacy, University of Porto, 4050-313 Porto, Portugal; jorge.emt.soares@gmail.com (J.S.); mcamorim@ff.up.pt (C.R.-P.); 2 Associate Laboratory i4HB—Institute for Health and Bioeconomy, Faculty of Pharmacy, University of Porto, 4050-313 Porto, Portugal; 3 TOXRUN—Toxicology Research Unit, University Institute of Health Sciences, CESPU, CRL, 4585-116 Gandra, Portugal; 4 Department of Pharmacology and Toxicology, Faculty of Pharmacy, Charles University, 500 05 Hradec Králové, Czech Republic; mladenkap@faf.cuni.cz

**Keywords:** cathinone, cathine, kinetics, toxicology, amphetamine-like, norpseudoephedrine

## Abstract

Khat (*Catha edulis*) is a recreational, chewed herbal drug that has been used as a psychostimulant for centuries in East Africa and the Arabian Peninsula, namely in Somalia, Ethiopia, and Yemen. However, the growing worldwide availability of khat has produced widespread concern. The plant comprises a large number of active substances, among which cathinone, cathine, and norephedrine are the main constituents, which can be included in the group of sympathomimetics of natural origin. In fact, these compounds are amphetamine analogues, and, as such, they have amphetamine-like nervous system stimulant effects. Chewing the leaves gives people a sensation of well-being and increases energy, alertness, and self-confidence. The chronic use of khat is, however, associated with severe cardiac, neurological, psychological, and gastrointestinal complications. The psychological dependence and withdrawal symptoms of khat are the reasons for its prolonged use. The aim of this paper is to review current knowledge on the khat plant with toxicokinetic and toxicodynamic perspectives. Namely, this review paper addresses in vitro, in vivo, and human studies. The models used, as well as the concentrations and doses with the respective biological effects, are discussed. Additionally, the main drug interactions involved with khat are described.

## 1. Introduction

Khat (*Catha edulis*, *Celestraceae*) is an evergreen shrub or tree (1–25 m tall), the fresh leaves of which contain cathinone, cathine, and norephedrine, which are sympathomimetic compounds. The plant is native to the Horn of Africa and the Arabian Peninsula and has been widely cultivated and consumed for centuries due to its psychostimulant effects [1,2]. Khat use for medical purposes is documented in the New Testament: the plant was considered as “divine food”, whereas Egyptians used it to achieve “apotheosis”—the feeling of transformation into a “god-like” stature. *Kitab al-Saidana fi al-Tibb* is the oldest known (11th century) pharmaceutical and medical description of khat [2]. In 1775, during a trip to Egypt and Yemen, botanist Peter Forskal identified the plant as *Catha edulis,* a member of the family *Celastraceae*. Other local names for *Catha edulis* are qat, q’at, kat, kaht, gat, chat, tschat, mira, and murungu [2,3]. A famous Ethiopian folklore story tells that the first human user of this plant was a Yemeni shepherd, who noted the effects of khat on his goats. After the discovery of its effects, khat use became widespread between the occult, spiritual, and upper classes in Yemen in the 16th century. Afterward, in the beginning of the 19th century, its use turned out to be extraordinarily broad and practically universal in certain parts of Yemen [4].

Fresh khat leaves are chewed by more than 20 million people across the Arabian Peninsula and East Africa every day, and this habit is deeply rooted in the socio-cultural traditions of these countries [5]. In Yemen, 60% of males and 35% of females are daily khat chewers. In a social khat session in Yemen, a quantity of 100–200 g of fresh leaves is usually consumed [4]. Khat is sold in markets and is presented as a package of twigs, stems, and leaves wrapped in banana leaves to maintain its freshness [2]. This tendency has spread to ethnic communities in the rest of the world, such as the Somali communities in South Wales and London. In the US, its use is more common among Yemenis, Somalian, and Ethiopian immigrants [6]. The amount of khat confiscated in Europe has increased practically ten-fold since 2001, in particular in France and Germany [7].

Despite previous reviews on the analytical points, chemical issues, and pharmacological side effects of khat [6,8,9,10], there is a need for an updated review focusing on the sympathomimetic action, toxicokinetics, and toxicodynamics of the active principles of khat. This paper intends to review the khat plant with this perspective.

## 2. Results and Discussion

### 2.1. Khat Phytochemistry

**Figure 1:**
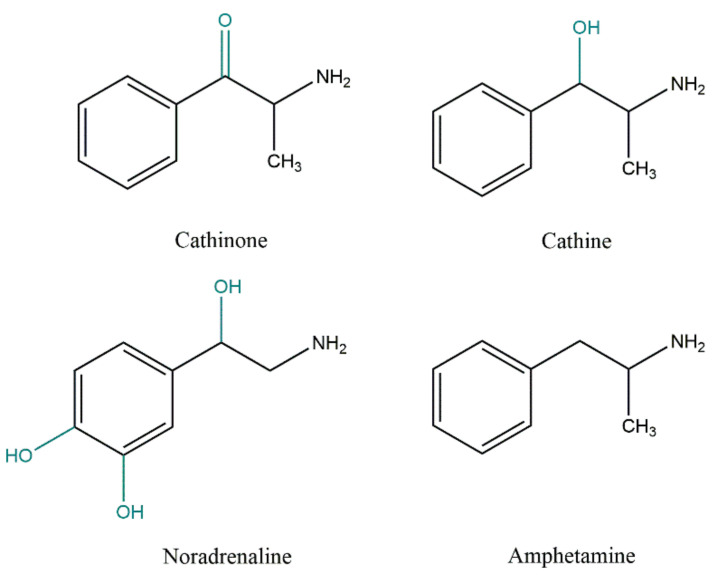
Chemical structures of cathinone, cathine (norpseudoephedrine), noradrenaline, norephedrine, and amphetamine. Structural differences to amphetamine are underlined (blue) in the other compounds.

Wolfes found norpseudoephedrine (cathine) in the leaves of khat in 1930, and this alkaloid was considered to be the major active ingredient of khat until the 1960s. However, due to the statement of Brücke in 1941 that the amount of this alkaloid was not enough to account for the khat users’ symptoms, the chemistry of the plant was further studied, and cathinone was isolated for the first time [8,9]. In fact, cathinone is one of the most abundant alkaloids in fresh leaves of *Catha edulis,* and the principal active constituent responsible for the stimulant effects of this ‘natural amphetamine’ [11]. Nevertheless, cathinone is relatively unstable and decomposes into cathine and norephedrine after harvesting. This process is potentiated when the leaves are dried. Thus, only freshly picked leaves have full efficacy [10,12]. On average, fresh khat is reported to contain 36–343 mg of cathinone [3,13], but also 83–120 mg of cathine and 8–47 mg of norephedrine per 100 g of leaves [13]. These compounds are structurally (Figure 1) and pharmacologically similar to amphetamine and noradrenaline, showing their potential to affect the central and peripheral nervous systems [14].

Other phenylalkylamine alkaloids, such as phenylpentenylamines, merucathinone, pseudomerucathine, and merucathine, and also cathedulin alkaloids, are found in khat leaves, although their concentrations are relatively low [15]. However, the chemical profiles of khat leaves are dependent on the environment, climate conditions, and cultivation and harvesting processes. Overall, fresh khat leaves comprise numerous distinct chemical substances, which include the following: alkaloids, terpenoids, glycosides, sterols, tannins, and flavonoids; several amino acids, such as tryptophan, glutamic acid, glycine, alanine, and threonine; vitamins, comprising ascorbic acid, thiamine, riboflavin, niacin, and carotene; elements, such as calcium, iron, manganese, copper, and zinc; and toxic metals, such as lead and cadmium [15,16,17].

### 2.2. Khat Legality

Cathinone has been listed in Schedule I of the Controlled Substances Act since 1993, while cathine was listed as a Schedule IV substance in 1988 [3]. Khat itself is not included in the controlled substances list, and, therefore, the legal status of the plant itself is often challenging and can differ among different countries [7]. Nevertheless, the khat plant can be placed into Schedule I when containing detectable levels of cathinone.

**Figure 2:**
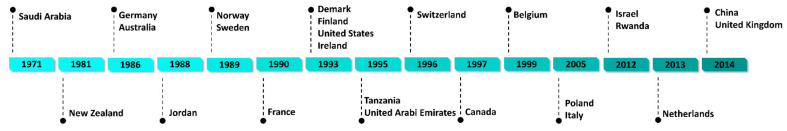
Historical sequence of the countries in which khat became a controlled substance.

Countries all over the world have gradually treated khat as a controlled substance (Figure 2). Khat is currently controlled in many countries in Europe, Asia, and North America [7]. However, it is still legal in most East African countries [3]. It has become Ethiopia’s biggest export commodity after coffee. Over the last two decades, the extent of land allocated to khat production has increased by 160%, with hundreds of millions of kilograms of khat produced yearly [7].

### 2.3. Khat Toxicokinetics

Khat is typically chewed, sporadically infused as a tea, and rarely smoked [15]. Approximately 100–500 g of khat leaves are chewed in a single khat session over several hours. When chewed, the leaves are detached from their branches and kept in the mouth (chewed sporadically to discharge the active principles, or kept in the oral cavity) and later expectorated [5,18]. Approximately 90% of the alkaloids are effectively extracted from the leaves into the saliva by chewing [19].

Following khat chewing, the psychostimulant effects appear 30 min later and last for 3 h [3]. The oral mucosa performs a major role in the absorption of compounds from khat (60% of total absorbed. After swallowing, further absorption occurs in the stomach and small intestine [15,19,20]. The maximum plasma concentration of cathinone is dependent on the dose ingested and is achieved within 1.5–3.5 h. Table 1 summarizes the maximum plasma concentrations and terminal half-lives found in human volunteers after khat chewing.

**Figure 3:**
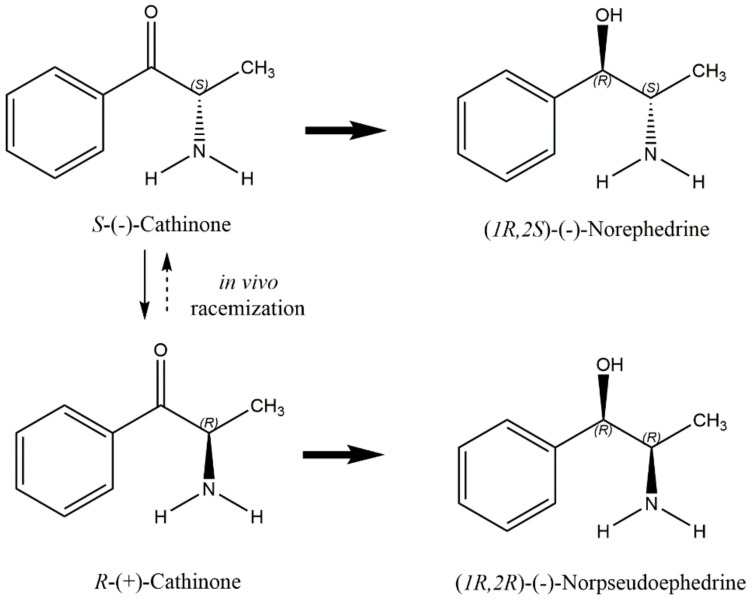
Stereoselective cathinone metabolism and the main metabolites excreted in urine (adapted from [24,25]).

Cathinone undergoes phase I metabolism, catalysed by liver microsomal enzymes, namely via reduction of the β-keto group to an alcohol, producing cathine and norephedrine (Figure 3) [24,25]. Cytochrome P450 2D6 (CYP2D6) seems to be involved in the conversion of cathinone to cathine [26]. The metabolism is stereoselective: the major metabolite of *S*-(−)-cathinone is norephedrine (*R*,*S*-(-)-Norephedrine), whereas the major metabolite of *R*-(+)-cathinone is cathine (*R*,*R*-(-)-Norpseudoephedrine). Overall, almost all the cathinone is quickly and stereoselectively metabolized, and only a small amount, lower than 7%, is excreted unchanged in the urine [24,25].

### 2.4. Khat Toxicodynamic

Cathinone and cathine are responsible for the main psychoactive and sympathomimetic effects of khat. Although both of them can stimulate the central nervous system (CNS), cathinone is mainly responsible for all the initial CNS actions of khat. Cathinone is a β-keto analogue of amphetamine (Figure 1), and, *per se*, it has amphetamine-like CNS stimulant effects [16,27,28]. Amphetamine derivatives belong to the class of drugs called the ‘β-phenylethylamines’, and they are structurally similar to catecholamine neurotransmitters, noradrenaline and dopamine. The structural analogy between amphetamine derivatives and noradrenaline explains their sympathomimetic activity. Moreover, amphetamine presents similarities to ephedrine, having norephedrine as a common metabolite with cathinone. Amphetamine derivatives are competitive substrates for monoamine reuptake transporters. Additionally, amphetamine acts on the CNS as a monoamine releasing agent (noradrenaline, dopamine, and serotonin) and releases adrenaline from the peripheral sympathetic nervous system [29]. This mechanism is accompanied by monoamine reuptake inhibition and possibly also monoamine oxidase (MAO) inhibition, consequently augmenting synaptic monoamine concentrations.

**Figure 4:**
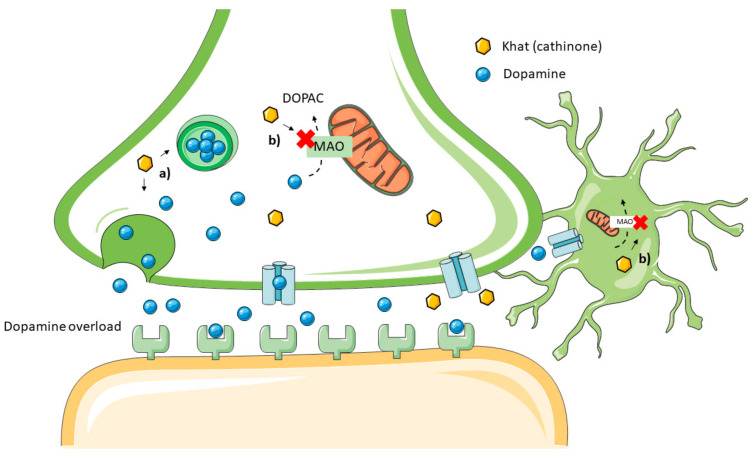
Mechanism of toxicity of cathinone on the central nervous system. (**a**) Dopamine release induction, (**b**) MAO inhibition in neurons and astrocytes.

Indeed, cathinone has CNS stimulant and sympathomimetic effects, which are shared with other sympathomimetic and CNS stimulant compounds of both natural and synthetic origin (e.g., cocaine, amphetamines) [30]. Initial studies showed that cathinone is able to induce amphetamine-like CNS dopamine release [12] and is able to inhibit MAO, with a preference towards MAO-B, the inhibition of which leads to diminished dopamine degradation and subsequent synaptic accumulation [31,32]. These major mechanisms of action are shown in Figure 4. The increased stimulation of dopaminergic pathways in specific areas of the brain is associated with the euphoric effect of khat [16,27,33].

### 2.5. Addiction

Under normal conditions, khat chewing induces only limited psychotropic activity with a relatively short duration. Therefore, normally, khat triggers moderate psychological dependence, with no evident physical dependence [28]. Along with psychological dependence, the withdrawal symptoms of khat are bases for its continued use [1,27]. In East African countries, the dependence on this plant was estimated to be 5–15% of the population [34].

The use of khat normally begins at a young age and can grow into a long-lasting regular pattern [35]. Continued use depends on reinforcing psychostimulant action and deeply rooted cultural factors [15]. The psychostimulant action of khat is known to be due to an increase in levels of dopamine in the brain, caused by cathinone acting on the catecholaminergic synapse [35]. Chronic use of khat is often compulsive, as revealed by the consumers’ preference of a daily supply of the leaves instead of vital needs [12]. Khat has the potential to induce a higher dependence in users than amphetamine because of its less aversive nature and rapid onset of action [14]. Khat chewing is followed by lethargy, anergia, unpleasant dreams, slight trembling, and depression [15]. A study performed by Widmann et al. (2014) on khat chewers reported tolerance effect (66.7%), withdrawal symptoms (94.0%), additional uses than intended (72.7%), desire to reduce or stop consumption (78.8%), decreased social life (97.0%), and consumption regardless of health complications (93.9%) [36]. Male khat chewers revealed more khat dependence symptoms compared to females, while a correlation between age and dependence was observed in women [37]. Later, in 2016, Nakajima et al. [38] studied khat dependence in 270 khat users in Yemen, and the observed prevalence rate in male subjects in this research was 48.9%, which was consistent with the 51% reported in the UK [39], 52% in Saudi Arabia [40], and 44% in Australia [41].

### 2.6. Effects after Chewing Khat Leaves

#### 2.6.1. In Vitro Studies

The cytotoxic effects of fresh khat extract were previously evaluated by Al-Ahdal et al. (1988) on three types of cells (human epidermoid carcinoma cells (KB), normal human fibroblasts (1BR.3), and xeroderma pigmentosum fibroblasts (XP2Bi)), using a dose range of 0–80 ng/mL. A Lethal Dose 50% (LD_50_) of 40 ng/mL was reported in KB carcinoma cells, which exhibited little resistance to the extract at low doses. Contrariwise, the two fibroblast cell lines showed biphasic survival. An LD_50_ of 20 ng/mL was achieved for 25% of the cell population and 75 ng/mL for the more resistant subpopulation. Because of the established mutagenicity of khat extract, DNA synthesis was assessed and was shown to be inhibited by 50% at 200 ng/mL in KB cells, 45 ng/mL in 1BR.3 cells, and 60 ng/mL in XP2Bi cells [42].

Nyongesa et al. [43] studied testicular interstitial cell viability and levels of testosterone after incubation of isolated mouse interstitial cells with different concentrations of khat extract (0.06 to 60 mg/mL) over 3 h. The highest concentrations tested (30 mg/mL and 60 mg/mL) significantly inhibited testosterone production and decreased cell viability, while the low concentrations significantly stimulated testosterone production and had no effect on interstitial viability.

Dimba et al. (2004) studied the effects of organic khat extract on human leukaemia cell lines (HL-60, Jurkat, and NB4) and primary peripheral leukocytes over 8 h. This research showed that organic khat extract produced a consistent type of cell death in all tested models, exhibiting all the characteristics of apoptotic cell death. The apoptotic cell death induced by khat occurred through mechanism(s) that were regulated by the activation of cellular caspase −1, −3, and −8 [44]. With these results as a basis, Bredholt et al. (2009) tested the effects of organic khat extract and the topoisomerase I inhibitor, camptothecin, on acute myeloid leukemia (AML) cell lines. AML cell lines were exposed to 200 μg/mL khat and 0.1 and 1.0 μM camptothecin for 8 h. Khat initiated a distinct cell death pathway compared to camptothecin, including mitochondrial damage and autophagy [45].

Abou-Elhamd et al. (2021) assessed the effects of khat at different concentrations, ranging from 10 μg/mL to 10 mg/mL on a human ovarian adenocarcinoma cell line (SKOV3) for 24–72 h. Khat generated diminished cell size, cell membrane injury, and apoptosis. At high concentrations, cell metabolic activity, cell cycle, and cellular proliferation were impacted. In silico study suggested that khat constituents (cathine, cathinone, and catheduline) bind to family A of G-protein-coupled receptors. Furthermore, significant players (cAMP-response element binding protein (CREB), fibroblast growth factor (FGF), Interleukin 6 (IL-6), extracellular signal-regulated kinase 1/2 (ERK) and mitogen-activated protein kinase (MAPK)) implicated in endometrial cancer and the cell cycle were altered [46]. Table 2 recaps all the studies cited above.

#### 2.6.2. Human Studies

The effects felt by a khat chewer can be distributed into desirable (in the first hour) and undesirable (at the end of the desirable effects and continue for some hours) [4].

Khat chewers’ reports describe sensations of happiness, improved energy, excitement, euphoria, alertness, enhanced self-esteem, increased ability to concentrate, increased libido, enhanced imaginative ability, improvement in communication ability, capacity to associate ideas, and subjective improvement in work performance. Nevertheless, chewers can also experience some negative experiences, such as over-talkativeness, over-activity, wakefulness, irritability, anxiety, hostility, psychotic illness, hysteria, and depression [47]. Prolonged use of khat is usually followed by cardiac, neurological, psychological, and gastrointestinal problems [3]. Khat use produces increased heart rate and blood pressure in humans due to the indirect sympathomimetic activity of cathinone, persisting for around 3–4 h after use [19,21]. According to Tesfaye et al. (2008), Ethiopian individuals that chew and smoke khat are linked with significantly elevated diastolic blood pressure [48]. In addition, there is evidence of enhanced risk of myocardial infarction (MI) and cardiac arrhythmias [49,50]. Khat chewing is also correlated with sweating, palpitation, and cold peripheral extremities [51].

The astringency of tannins present in khat leaves can cause oesophagitis, gastritis, and oral mucosal keratosis. Reports have shown that about 50% of khat chewers develop oral mucosal keratosis [52], which may develop into oral cancer [53]. After khat consumption, cathinone also has appetite suppressant effects, acting centrally in the hypothalamus, causing delays in gastric emptying [6,54,55].

Long-term users of khat usually develop complications, such as acute and chronic liver disease. Chronic khat use can lead to hepatitis, fibrosis, and cirrhosis [56,57]. 

Khat use also affects human reproductive health. Sperm count and motility were reported to be lower amongst addicts [47,58]. In addition, Khat has teratogenic effects in pregnant women [58].

Khat, in high doses, can induce manic illness with grandiose delusions or schizophreniform psychosis with persecutory delusions. In the majority of cases, the symptoms quickly stop when khat is withdrawn and antipsychotic medications are used. However, there is a predisposition for the reappearance of psychotic episodes upon resumption of khat use [59,60,61,62].

**Figure 5:**
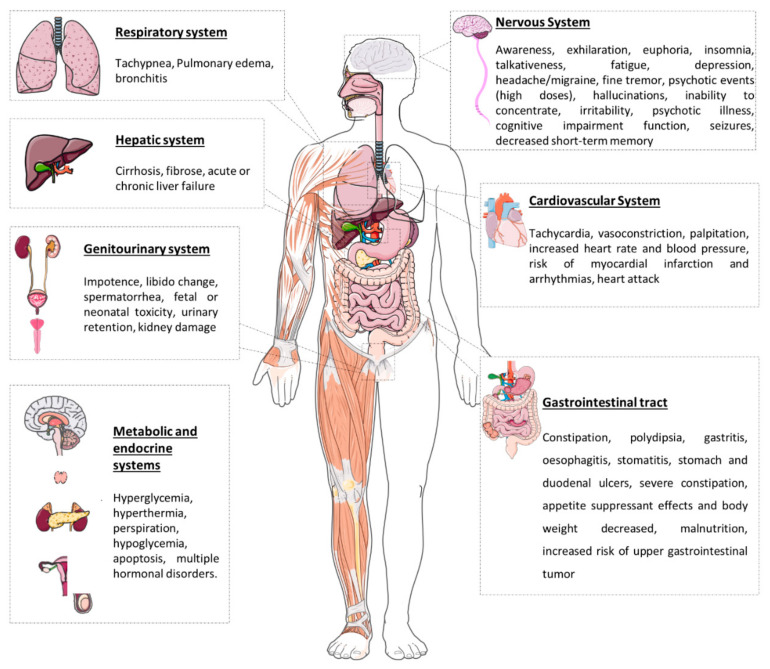
Common adverse effects of khat abuse [1,3,26,51].

The short- and long-term physical effects of khat consumption have been well described according to the physiological systems involved and are summarized in Figure 5.

### 2.7. Khat Interactions

The adverse effects of khat can be particularly intensified by the concomitant use of some conventional drugs. These interactions can be synergistic or inhibitory. Several studies have reported interferences with anaesthetic drugs and also reductions in the bioavailability of antibiotics caused by khat chewing [63,64,65,66,67]. Table 3 summarizes the main results of these studies.

Due to khat mechanism(s) of action, some other drug interactions are expected to occur. As mentioned above, studies performed on animals and humans demonstrated that khat or cathinone can increase blood pressure. Therefore, it is expected that khat could counteract the effects of co-administered antihypertensive drugs and develop some resistance to drug treatment. Since MAO inhibitors are a group of drugs that increase synaptic concentration of monoamines, in the presence of khat, MAO inhibitor effects are likely to improve, as cathinone has also been shown to act both as a MAO inhibitor and as an inducer of monoamine release. Therefore, concurrent use of MAO inhibitors with khat should be avoided. The same outcome is likely for the simultaneous use of CNS stimulant drugs and khat, since it is predicted to have additive or synergistic effects. A recent study performed in human volunteers showed that khat inhibited the metabolic activity of CYP2D6, an enzyme involved in the metabolism of several therapeutic drugs [68]. Thus, the concomitant use of khat with drugs that are substrates of CYP2D6 can lead to drug overdoses.

## 3. Conclusions

Khat (*Catha edulis*) is a chewable, herbal psychostimulant composed of cathinone, cathine, and norephedrine, the first two being primarily responsible for the observed psychoactive and sympathetic effects. Khat constituents are easily absorbed in the digestive tube, with cathinone being quickly and stereoselectively metabolized to cathine and norephedrine. Their effects are predominantly mediated by monoamine release and reuptake inhibition, and possibly also in part by inhibition of MAO. These mechanisms of action combine additively or synergistically to augment synaptic monoamine concentrations.

As amphetamine analogues, they provide happiness and increase energy, alertness, and self-esteem. These desirable effects are felt during the first hours of khat use, while the undesirable effects start near the end of the desirable effects and continue for some hours. In addition to the acute negative side effects, chronic use of khat causes psychological dependence and withdrawal effects, as well as severe cardiac, neurological, psychological, and gastrointestinal effects, between other complications. Indeed, several khat studies have demonstrated its harmful effects. A negative impact on reproductive health (e.g., cathinone increased aggressivity and altered sexual behaviour), with embryotoxic and teratogenic effects (e.g., retardation of growth rate), were described. Khat-associated cardiovascular toxicity was also observed, namely vasoconstriction and positive chronotropic effects, thus explaining the concomitant increase in arterial blood pressure. Consequences include an increased incidence of MI and cardiac arrhythmias. Furthermore, the renal and immune systems can also be negatively affected by khat. The astringency of tannins present in khat leaves can cause esophagitis, gastritis, and oral mucosal keratosis, which can develop into oral cancer. Liver disease was reported in khat users. In summary, khat misuse is associated with many several adverse reactions, both in acute and chronic settings, and might also alter the activity of several concomitantly used approved drugs.

## Figures and Tables

**Table 1:** Maximum plasma and terminal half-life found in plasma of healthy human volunteers after khat chewing.

Subjects	Oral Cathinone Dose (per Kilogram of Body Weight)	Maximum Plasma Concentration	Terminal Elimination Half-Life	Ref.
Six male volunteers (25–35 years)	0.8 mg (54–71 g of fresh khat leaves)	127 ± 53 ng/mL after 2.1 ± 0.5 h	4.3 ± 1.7 h	[21]
Five volunteers (two females and three males) (21–30 years)	0.8–1 mg (60 g of fresh khat leaves)	83 ± 42 ng/mL after 1.5–3.5 h	-	[22]
Four volunteers (two male and two female) (26–57 years)	0.6 mg (26–59 g of fresh khat leaves)	58.9 ± 18.8 ng/mL after 2.31 ± 0.65 h	1.5 ± 0.8 h	[19]
Six male volunteers (28–36 years)	0.5 mg	1 h	-	[23]

**Table 2:** In vitro studies of khat.

**In Vitro Model**	**Concentration of** **Khat Extract**	**Results**
KB cells [42]	0–80 ng/mL	LD_50_ of 40 ng/mL DNA synthesis inhibition by 50% at 200 ng/mL
1BR.3 and XP2Bi [42]		Biphasic survival (LD_50_ of 20 ng/mL for 25% of the cell population and 75 ng/mL for the more resistant subpopulation) DNA synthesis inhibition by 50% at 45 ng/mL in 1BR.3 cells, and 60 ng/mL in XP2Bi cells
Mouse interstitial cells [43]	0.06, 0.6, 6, 30 and 60 mg/mL	The highest concentrations (30 mg/mL and 60 mg/mL): significantly inhibited testosterone production and decreased the cell viability The lowest concentrations (0.06, 0.6, and 6 mg/mL): significantly stimulated testosterone production and had no effect on interstitial viability
HL-60, Jurkat, NB4 cell lines, and primary peripheral leukocytes [44]		Organic khat extract induced apoptotic cell death, regulated by the activation of cellular caspase −1, −3, and −8
MOLM-13, MOLM-14, NB4 and MV-4-11 cell lines [45]	200 μg/mL	Organic khat extract activated a distinct cell death involving mitochondrial damage and morphological features of autophagy
SKOV3 [46]	0.01, 0.03, 0.1, 0.3, 1, 3, and 10 mg/mL	Khat induces reduced cell size, cell membrane damage, and apoptosis The highest concentrations (1, 3, and 10 mg/mL) affected cell metabolic activity, cell cycle, and cellular proliferation
In silico [46]		Khat constituents (cathine, cathinone, and catheduline): bound to family A of G-protein-coupled receptors and altered several signalling pathways (CREB, Wnt, FGF, IL-6, and ERK/MAPK)

**Table 3:** Pharmacological interactions between khat and clinically used drugs.

Drug Classes	Drug	Results
Anaesthetics	benoxinate (0.4%) [65]	Consumption of khat (12 h prior surgery): Reduces pain tolerance and comfort of patients during local anesthesia and surgery
	sevoflurane (2%) plus nitrous oxide (65%) [66]	Consumption of khat (4 h prior surgery): Recovery from anesthesia was delayed
Antibiotics	ampicillin (500 mg); amoxicillin (500 mg) [64] cephradine (500 mg) [63] tetracycline (500 mg) [67]	The astringent activity of tannins present in khat on GI surface can prevent/reduce absorption of oral drugs The bioavailability of both drugs was significantly reduced by khat Reductions in maximum plasma concentration, slower time for reaching peak concentration, and decrease in absorption rate

GI, gastrointestinal.

## Data Availability

No new data were created or analyzed in this study. Data sharing is not applicable to this paper.
